# Gender Inequality in Managing Childhood Sleep: Which Parent Gets up at Night?

**DOI:** 10.3390/children12040491

**Published:** 2025-04-10

**Authors:** Agnès Breton, Florian Lecuelle, Louise Chaussoy, Madeleine Heitz, Wendy Leslie, Royce Anders, Marie-Paule Gustin, Patricia Franco, Benjamin Putois

**Affiliations:** 1 Faculty of Psychology, Unidistance Suisse, 3900 Brig, Switzerland; agnes.breton@etu.unidistance.ch (A.B.); florian.lecuelle@chu-lyon.fr (F.L.); madeleine.heitz@gmail.com (M.H.); 2 Lyon Neuroscience Research Center, CNRS UMR 5292-INSERM U1028, University of Lyon 1, 69000 Lyon, France; 3 Department of Pediatric Clinical Epileptology, Sleep Disorders and Functional Neurology, University Hospitals of Lyon, 69000 Lyon, France; 4 CeRCA, University of Poitiers, University of Tours, 86000 Poitiers, France; chaussoylouise@gmail.com; 5 Xceltranslate, 06000 Nice, France; wendyleslie06@gmail.com; 6 Université Paul Valéry Montpellier 3, EPSYLON EA 4556, Rte de Mende, 34000 Montpellier, France; royce.anders@univ-montp3.fr; 7 Public Health, Epidemiology and Evolutionary Ecology of Infectious Diseases (PHE3ID), International Centre for Infectiology Research (CIRI), INSERM U1111, CNRS UMR5308, ENS Lyon, 69000 Lyon, France; marie-paule.gustin@univ-lyon1.fr; 8 Institute of Pharmaceutic and Biological Sciences, Public Health Department, Biostatistics, University Claude Bernard Lyon 1, 69100 Villeurbanne, France

**Keywords:** gender disparities, father’s involvement, child sleep, child insomnia

## Abstract

Objective: Parental responsibilities for childcare remain unequally distributed between mothers and fathers. This study investigates whether such gender disparity also applies to night-time care, particularly when children experience sleep disorders. Methods: We conducted a cross-sectional survey study including 882 clinical files from sleep consultations for children aged 0 to 5 years, completed by one parent (98% mothers). To assess inter-rater reliability, 112 father–mother dyads outside the clinical setting were also surveyed. Additionally, 1409 mothers from the general population formed a control group. Results: In the clinical group, 60% of children were cared for exclusively by their mother at night, versus 9% by the father. In the control group, the figures were 64% and 6%, respectively. Gender disparities persisted even when both parents worked full-time or when the child was no longer breastfed. Inter-rater reliability was strong (r > 0.70). Factors such as number of night awakenings, child’s age, and maternal education influenced caregiving distribution. Greater maternal involvement was associated with increased psychological distress and lower relationship satisfaction. Conclusions: Mothers remain the primary caregivers at night, even in dual-earner families. This unequal distribution can affect maternal well-being and couple dynamics. Promoting paternal involvement may reduce maternal overload and improve child sleep outcomes.

## 1. Introduction

Prevalence of sleep disorders in children peaks before the age of 5 years: nearly 40% sleep poorly [1,2]. The term “childhood insomnia” is used in this study to describe difficulties in initiating and maintaining sleep (DIMS), characterized by repeated struggles in falling asleep and staying asleep without parental intervention at least three times per week for a minimum duration of three months [3]. Pediatric insomnia is behavioral in origin in approximately 80% of cases [3]. Rapid and independent sleep initiation is a learned behavior shaped by parental practices. Behavioral insomnia in children is primarily linked to frequent or active parental interventions at bedtime and during night-time awakenings [4,5,6,7].

Children who are unable to fall asleep independently experience more frequent night-time awakenings and require greater parental intervention, thereby affecting the entire family’s sleep [8,9]. These sleep disorders are linked to a decline in both the quality and quantity of maternal sleep, as well as heightened stress and increased symptoms of maternal depression [10]. As a result, affected mothers experience greater fatigue, irritability, and difficulty managing daily parenting tasks [11]. 

To ensure consistency in our operationalization of childhood insomnia, this study utilizes the Sleep Disturbance Scale for Children (SDSC) [11,12] and Difficulty in Initiating and Maintaining Sleep (DIMS) [11,12] subscale as primary measures, which have been validated in previous pediatric sleep research. A score exceeding the SDSC threshold of 37/110 and a DIMS subscale score above 16/40 are considered indicative of clinically significant insomnia symptoms [12]. These measures ensure that the definition of childhood insomnia used in our study aligns with established pediatric sleep disorder frameworks.

Parental beliefs play an important role in maintaining frequent or active night-time parental interventions. The latter depend on parents’ perception of their beliefs about setting limits or responding to their child’s crying [4,9]. Cognitions associated with limits are said to have the greatest impact on children’s sleep: the more parents find it difficult to set limits, the more frequent night-time awakenings will be [4]. Fathers have an important role to play in mother–child night-time separation [13,14]. On the one hand, they are biologically less sensitive than mothers to their child’s crying, they react less quickly to night-time awakenings, and tend to adopt a limit-setting approach to encourage self-soothing [7,13,15]. Involving fathers therefore has a direct impact on preventing or treating childhood insomnia, with fewer night-time awakenings and fewer difficulties falling asleep [13,14]. On the other hand, fathers’ night-time involvement has an indirect impact on the family’s overall well-being [5], limiting the risk of post-partum depression [16] and improving mothers’ sleep [14].

Day-time child-rearing is still mainly managed by mothers [17]. In 2010, 65% of parental tasks fell to women, and parenting time increased with the number of children for women only. This disparity also concerns domestic work, on which women spend 183 min a day, compared to 105 min for men. Women handle 73% of the cooking, 85% of the laundry, 93% of the ironing, 77% of the housework, 74% of the childcare, and 73% of the schoolwork [18]. Concerning child sleep, only one survey [19] has studied 4–20 year olds, and it showed male–female disparity: 31% of children were looked after by their mothers exclusively, compared with 16% by their fathers. Given that the prevalence of sleep disorders is highest before the age of 5 years, and that we can assume teenagers require less night-time intervention from their parents, the only survey available to date does not focus on the relevant age category. Are children under 5 years old more likely to be looked after at night by their mothers than their fathers? Biological and cultural arguments substantiate this investigation.

Biological factors are partly linked to night-time breastfeeding, recommended by the WHO up to twelve months of age to ensure optimal development. Breastfeeding is associated with less paternal involvement at bedtime [20,21], as fathers feel less invested in this feeding method [20]. Night-time breastfeeding is also associated with longer and more frequent night-time awakenings [8,21] and more crying at night. In addition to feeding-related arousals, a poorly conditioned infant who falls asleep at the breast will also require their mother’s presence for sleep initiation or for any night-time arousal, including non-feeding arousals. Beyond the first six months, this biological explanation only applies in the case of a night-time feeding syndrome [22]. We hypothesize that night-fed children are looked after more by their mothers than their fathers. This difference should theoretically diminish when the child is weaned at night.

Previous studies have shown that sleep duration and quality are not only biologically determined but also shaped by social and professional constraints [23]. In particular, the presence of young children disproportionately affects maternal sleep, reinforcing existing gender disparities in night-time caregiving.

Socio-economic and cultural dimensions could also explain the difference between men and women when it comes to looking after children at night. In the past, it was generally accepted that fathers were responsible for providing for the family’s financial needs, while mothers were responsible for raising the children and managing sleep, in order to give the father the time to rest after a day’s work. This patriarchal model is based on traditional conceptions, but no longer corresponds to today’s reality, given that the proportion of working women has risen steadily since the 19th century. However, despite this evolution in women’s participation in the workforce, inequalities persist. For example, while the rate of part-time work among women increases with the number of children, it remains relatively stable among men [18]. Does the professional occupation of fathers and mothers determine who looks after the child at night? We can hypothesize that fathers who work the same amount as mothers, or those who work less, are more involved in night-time care.

The greater involvement of mothers could also be explained by family composition: 13.2% of children under 3 live in a single-parent family, and in 82% of such cases, they live with their mother [18]. In these situations, the burden of night-time care falls almost exclusively on the mother, whether she is active in the labor market or not. This reinforces the idea that the greater involvement of mothers in children’s night-time care is influenced by historical, economic, and family factors.

However, although this inequality in the distribution of night-time care is frequently observed in clinical practice, it has not yet been the subject of objective research in the scientific literature, and to date, we have no data concerning the distribution of night-time care between fathers and mothers.

The distribution of parental responsibilities in managing nocturnal awakenings cannot be understood without considering several contextual factors. Among these, couple conflicts, parental day-time involvement, household task distribution, marital satisfaction, and disagreement over educational values are key elements.

Marital conflicts have been identified as a factor influencing parental mental load and the management of domestic tasks, including night-time care [10]. Unequal parental involvement could generate tensions between partners and impact marital satisfaction. Moreover, perceptions of educational values play a crucial role in parental involvement: parental disagreements on child education and care can influence the dynamics of nocturnal caregiving [24]. Thus, these variables were included in our study to explore their relationships with the distribution of night-time childcare responsibilities.

Additionally, maternal depression and symptoms of anxiety may play a key role in night-time caregiving. Previous research has shown that a mother’s disproportionate involvement in managing their child’s sleep is associated with increased stress and psychological distress [5]. These variables were incorporated into our study to analyze their potential impact on the division of night-time parental tasks and the overall well-being of mothers.

The main objective of this study is to quantify the distribution of responsibilities between parents with regard to sleep education and the management of sleep disorders in young children. Particular attention is paid to children who sleep poorly and therefore require frequent parental intervention. The secondary objective is to identify the factors influencing this distribution of night-time care between parents, including the child’s gender and age, rank in the sibling group order, parents’ professional activity and level of education, night-time feeding, the presence or absence of sleep disorders, and the child’s sleep hygiene. Finally, this study explores associations between night-time parental involvement and variables in daily life and satisfaction within the couple, as well as psychopathological variables, with the aim of analyzing interactions between contextual factors, individual characteristics, and night-time care arrangements. These results will contribute to our knowledge of family dynamics in the management of young children’s sleep.

## 2. Materials and Methods

### 2.1. Participants

Our French sample includes a clinical group, a control group, and a father–mother dyad group for inter-rater agreement assessment.

*Clinical group*. Comprising 882 files from the *sommeilenfant.org* (accessed on 1 Janaury 2024) telepsychology institute (2019–2025), this group includes children aged 0–5 years referred for sleep disturbances. Data were collected from voluntary participants who consented to their use for the purpose of this research. Mothers played a predominant role, initiating consultations in 60% of cases and attending them alone in 77% of cases. Fathers completed questionnaires alone in only 2% of cases, while mothers were involved in 98% of cases. Consequently, only mother-completed questionnaires were retained.

*Control group*. This group includes 1409 participants aged 6 months to 5 years, recruited online by the Panelabs polling institute from the general population, regardless of sleep issues. To match the clinical group, questionnaires were also exclusively completed by mothers. Given that mothers are predominantly the ones completing questionnaires in clinical settings (98% of cases), and fathers alone participate in only 2% of consultations, we decided, for consistency, to include only mothers’ responses in the out-of-consultation (control) sample as well. This methodological choice ensures comparability between groups and reflects real-life participation patterns.

*Inter-rater group*. To assess whether maternal responses could reliably reflect shared parental perspectives, we conducted an inter-rater reliability study with an independent sample of 112 mother–father dyads of children aged 0 to 5 years old in pairs, enabling us to verify the degree of agreement between both parents on key variables. Parents completed the questionnaires on paper or by email, whether their child had sleep problems or not.

For our study, we sampled children according to five age categories: 0–6 months; 6–18 months; 18–30 months; 30–36 months; and 3–5 years old. These age categories were defined on the basis of the child’s sleep development phases and, in particular, the need or not for night-time feeding [25,26,27,28,29].

### 2.2. Procedure

The data collected were treated confidentially, in accordance with the CNIL regulations in force on the protection of personal data. The “Managing Childhood Sleep” project was approved by the French Individual Protection Committee (*Comité de Protection des Personnes* (CPP) EST II) under identification number 21.01.18.75329. This conveys ethical approval. Participants completed the surveys online (resulting in a 100% completion rate) after giving their consent for the data to be used. In the control group, participants received financial compensation of 7 euros. In the clinical group, participants received sleep advice, diagnostic screening, and referral to professionals qualified to treat their child’s sleep disorder.

### 2.3. Measures

A series of non-standardized questions was designed for the purpose of this study: The main outcome of this study was the answer to the question “Who looks after the child most nights?” on a 5-point scale (i.e., the mother all the time; most often the mother; both the father and the mother; most often the father; the father all the time). For the purposes of this study, we simplified this scale to three points: mainly mother, both parents, mainly father. Factors influencing the distribution of the caregiving role were measured using socio-demographic variables (age, marital status, occupation, and level of education) and child-related variables (age category, gender, rank in the sibling group order, feeding (breast or bottle), number of night-time awakenings, sleep disorders, insomnia, and organic disorders). Although parental night-shift work could potentially influence our findings, participants concerned by this factor (8 mothers and 14 fathers) were excluded from the present study due to their limited number. To better understand the influence of marital conflicts, parental day-time involvement, household task distribution, and marital satisfaction on night-time childcare, several measurement tools were used. Marital conflicts were assessed using a marital conflict scale that measures the frequency and intensity of disagreements between partners. Marital satisfaction was evaluated using the Marital Satisfaction Index, a validated measure for analyzing perceived relationship well-being. Household task distribution and parental day-time involvement were assessed through standardized questionnaires evaluating daily parental responsibilities. Lastly, disagreement on educational values was measured using a specific questionnaire on parenting practices. All self-reported measures were rated on a 0 to 100 scale, providing a continuous evaluation of each construct. This scoring system allows for precise differentiation of individual experiences and perceptions within a study.

A series of standardized questionnaires was used to measure psychopathological variables:

The Sleep Disturbance Scale for Children (SDSC) assesses sleep disorders in children aged 6 months to 4 years [11,12]. It includes 22 items rated by parents on a 5-point scale (“Never” to “Always”) and is divided into five subscales: hyperhidrosis, respiratory problems, parasomnias, non-restorative sleep and excessive day-time sleepiness, and difficulty in initiating and maintaining sleep (DIMS). The DIMS sub-score, focusing on sleep duration, latency, difficulties, and night-time awakenings, was central to this study. Sleep disorders are indicated by an SDSC score above 37/110, and DIMS issues by a score above 16/40. For analysis, 47 children under 6 months and 38 over 4 years were excluded.

The Sleep Hygiene Scale for Children (SHSC) is a French adaptation [11] of the Brief Infant Sleep Questionnaire (BISQ) [30], assessing child sleep hygiene across three factors: parental attachment, coping strategies, and screen exposure. Parental attachment focuses on conditions for falling asleep (e.g., breastfeeding or sleeping alone) and parental actions during sleep onset or night-time awakenings (e.g., removing the child from their bed). Behaviors promoting sleep disorders were selected to calculate a score out of 18.

The Parental Burnout Inventory (PBI) [31] includes 22 items evaluating emotional exhaustion, emotional distancing, and personal fulfillment. Responses are rated on a 7-point Likert scale, ranging from 0 (never) to 6 (everyday), with personal fulfillment items scored in reverse. A score above 88 indicates parental exhaustion syndrome.

The Beck Depression Inventory (BDI-13) [32] assesses maternal depression using a 13-item self-report questionnaire scored on a 4-point Likert scale (0–3). Scores range from 0 (no depressive symptoms) to 39, with symptom severity categorized as no depression (0–4), mild (4–7), moderate (8–15), and severe (16–39). In this study, a score above 15 indicated depression.

The State–Trait Anxiety Inventory (STAI-Y) [33] consists of two 20-item self-reported questionnaires (Y-A for state anxiety; Y-B for trait anxiety) measuring habitual anxious temperament. The French version uses a 4-point Likert scale, with total scores ranging from 20 to 80. Anxiety intensity is categorized as no/little anxiety (20–37), moderate anxiety (38–44), and severe anxiety (45–80). In this study, thresholds were set at 41 for the Y-A form and 44 for the Y-B form, with higher scores indicating anxiety.

The Insomnia Severity Index (ISI) [34] is a 7-item self-report questionnaire evaluating perceived insomnia severity over the past two weeks. Responses are rated on a 5-point Likert scale (0–4), with total scores ranging from 0 to 28. Insomnia is categorized as no insomnia (0–7), subclinical insomnia (8–14), moderate insomnia (15–21), and severe insomnia (22–28). In this study, a score of 15 or higher identifies clinically significant insomnia.

### 2.4. Statistical Analyses

*Inter-rater agreement* was evaluated for the question, “Who looks after the child most nights?”, as well as for total SDSC scores and the DIMS score using Pearson correlation analyses.

*Distribution of night-time care between fathers and mothers:* A chi-square analysis compared the gender distribution of the caregiver role in clinical and control groups. Pearson correlation analyses were conducted in the paired group to compare parents’ responses to “Who looks after the child most nights?”, total SDSC scores, and the DIMS score.

*Factors influencing the distribution of the caregiver role*: Multinomial logistic regression was used for the continuous variable “age of parents”. Chi-square analyses were applied to categorical variables, including professional occupation, parents’ education level, child’s age and gender, sibling rank, feeding method, night-time awakenings, presence of insomnia, and organic disorders. ANOVA analyzed the presence of sleep disorders (SDSC score) and the child’s sleep hygiene (SHSQ score). Due to variance heterogeneity, a Kruskal–Wallis test with Dunn’s post hoc tests and Bonferroni correction was applied for the variable “Who looks after the child most nights?”.

*Association with variables of daily life, satisfaction within the couple, and agreement on educational values*: ANOVAs analyzed parental day-time involvement, agreement on educational values, couple conflicts, and satisfaction within the couple. When ANOVA premises were unmet, Kruskal–Wallis tests with Dunn’s post hoc tests and Bonferroni correction were used. These analyses focused on the variable “Who looks after the child most nights?”.

*Association with psychopathological variables*: Simple linear regressions assessed the impact of the child’s insomnia (DIMS) on parental burnout (PBI), depression (BDI-13), anxiety (STAI), and insomnia (ISI). Pearson correlation analyses examined links between night-time awakenings and parental depression, burnout, and anxiety scores. Multiple linear regressions explored the relationship between the distribution of night-time parenting roles and parental burnout, depression, and anxiety. Chi-square analyses investigated relationships between parental insomnia, child insomnia, night-time awakenings, and the distribution of parental roles.

## 3. Results

The inter-rater agreement is high for “Who looks after the child most nights?” (r = 0.76, *p* < 0.01), total SDSC score (r = 0.70, *p* < 0.01), and DIMS score (r = 0.73, *p* < 0.01).

### 3.1. Night-Time Distribution of the Caregiver Role

Regarding the distribution of night-time care between mothers and fathers, a significant difference in caregiver role distribution was observed in the total sample (χ^2^(2) = 11.82, *p* = 0.003). In the clinical group, mothers were primarily responsible in 60% of cases, versus 31% sharing the task and 9% fathers. In the control group, mothers assumed 64% of responsibilities, with shared care at 30%, and fathers at 6%.

### 3.2. Parent-Related Factors of Influence

#### 3.2.1. Age of the Parents

No significant effect of parental age on caregiver role distribution (*p* > 0.05) (Table 1).

#### 3.2.2. Professional Occupation

**Figure 1:**
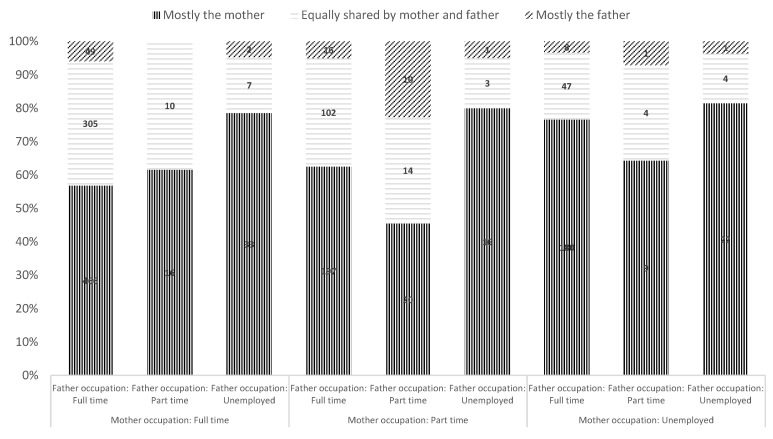
Paternal and maternal involvement in night-time care according to their professional activity, total sample.

Significant differences exist in night-time involvement based on professional activity (χ^2^(6) = 50.93, *p* < 0.001 for mothers; χ^2^(6) = 25.13, *p* < 0.001 for fathers) (Figure 1). Mothers predominantly handle night-time care regardless of employment status (Supplementary Data S1a). Fathers’ involvement remains minimal, even when unemployed or working part-time (Supplementary Data S1b).

#### 3.2.3. Parent’s Level of Education

Parental night-time involvement significantly varies with education (χ^2^(14) = 55.96, *p* < 0.001 for mothers; χ^2^(14) = 86.85, *p* < 0.001 for fathers). Mothers with lower education primarily take on care, especially in the clinical group (Supplementary Data S2a). Higher-educated mothers share care more often, particularly in the control group. Fathers with higher education show slightly increased involvement but remain negligible in the clinical group (Supplementary Data S2b). Whatever their level of education, or that of the father, mothers are mainly responsible for their children’s night-time care.

### 3.3. Child-Related Factors of Influence

#### 3.3.1. Child’s Age Category

**Figure 2:**
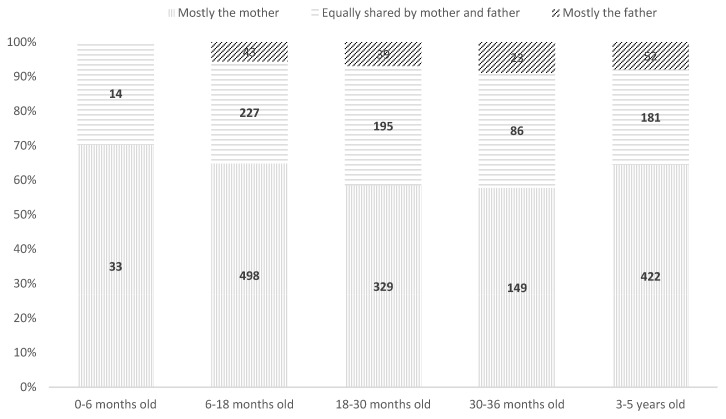
Distribution of night-time care between fathers and mothers according to child’s age, total sample.

Care distribution significantly varied by age category (χ^2^(8) = 17.42, *p* = 0.023) (Table 2). Shared care increased with age, peaking at 34% for children aged 18–30 months, though maternal dominance persisted (Figure 2).

#### 3.3.2. Gender and Childcare

No significant effect of child gender on care distribution (*p* > 0.05).

#### 3.3.3. Rank in the Sibling Group Order

Significant association found (χ^2^(6) = 23.11, *p* < 0.001), with mothers of only children more frequently handling care alone.

#### 3.3.4. Child Feeding Method

**Figure 3:**
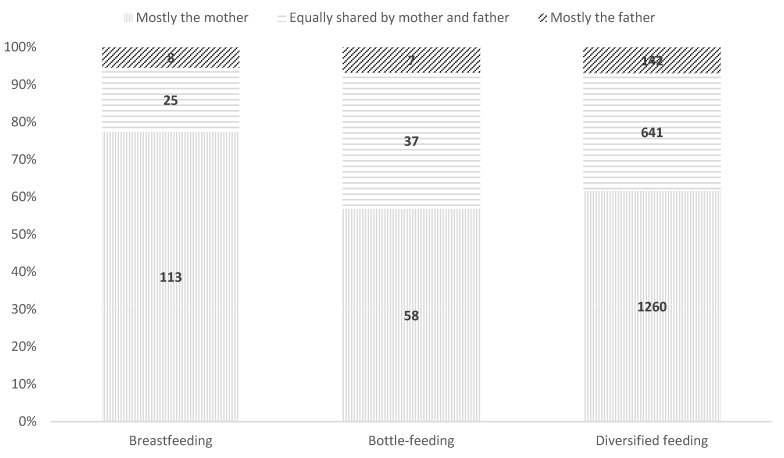
Distribution of night-time care between fathers and mothers according to child feeding method, total sample.

Significant differences were observed (χ^2^(4) = 16.47, *p* = 0.002) (Figure 3). Breastfeeding mothers were predominantly responsible, with minimal paternal involvement. The analysis of standardized residuals (Supplementary Data S4) shows no significant effect on fathers’ involvement in night-time care when bottle-feeding or diversified feeding is introduced. In both groups, mothers remain the primary caregivers, regardless of the child’s evolving dietary needs.

#### 3.3.5. Number of Night-Time Awakenings

**Figure 4:**
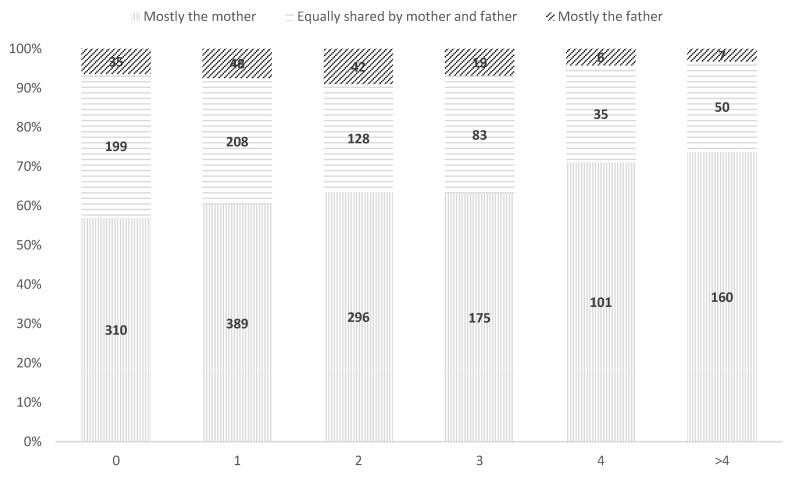
Distribution of night-time care between fathers and mothers according to the number of night-time awakenings, total sample.

Maternal involvement increased with more frequent awakenings (χ^2^(18) = 48, *p* < 0.001) (Figure 4). Shared care is higher with fewer awakenings. Analyses of standardized residuals (Supplementary Data S5) indicate that an increased number of night-time awakenings correlates with greater maternal involvement in both groups, and that co-parenting is particularly strong in the absence of awakenings.

#### 3.3.6. SDSC Score

There is no significant association between SDSC scores and care distribution (*F*(2, 2285) = 2.33, *p* = 0.098), indicating that the SDSC score does not vary according to which parent looks after the child at night. In addition, group affiliation does have a significant effect on sleep disorders (*F*(1, 2285) = 188.70, *p* < 0.001).

#### 3.3.7. DIMS Score

The analysis reveals no statistically significant association between the distribution of night-time care and presence of insomnia (DIMS) across the whole sample (χ^2^(2) = 3.68, *p* = 0.16). However, in the clinical group, a significant association is observed (χ^2^(2, N = 882) = 6.81, *p* = 0.03), where equal parental responsibility is linked to the absence of insomnia (DIMS < cutoff), and maternal care is predominantly associated with insomnia (DIMS > cutoff). No significant association is found in the control group (χ^2^(2, N = 141) = 2.87, *p* = 0.24).

#### 3.3.8. Organic Disorders in Children

No significant relationship between organic disorders and night-time care distribution (χ^2^(2) = 1.61, *p* = 0.45) was found, suggesting no impact on parental dynamics.

*Sleep hygiene*. SHSC scores were significantly associated with night-time care distribution (*F*(2, 2285) = 15.77, *p* < 0.001, η^2^ = 0.014). Scores were higher in the clinical group (M = 3.97) than in the control group (M = 1.57), *F*(1, 2285) = 202.54, *p* < 0.001, η^2^ = 0.081). Post hoc analyses revealed higher SHSC scores when mothers were primarily responsible compared to shared care (*p* = 0.039). No significant differences were observed for father-exclusive care. Elevated scores in the clinical group confirm less favorable sleep habits (*p* < 0.001).

### 3.4. Variables Relating to Daily Life and Satisfaction Within the Couple

#### 3.4.1. Responsibility for Children’s Day-Time Activities

Mothers who primarily handle night-time care also take on more day-time tasks (*F*(2, 2009) = 38.47, *p* < 0.001, η^2^ = 0.037). Parents in the clinical group assume significantly more daytime responsibilities than those in the control group (*F*(1, 2009) = 696.61, *p* < 0.001, η^2^ = 0.258), reflecting greater child support needs. An interaction effect (*F*(2, 2009) = 12.61, *p* < 0.001, η^2^ = 0.012) shows that in the clinical group, mothers bear an even greater day-time burden when night-time care is unevenly distributed.

#### 3.4.2. Responsibility for Domestic Tasks

Responsibility for domestic tasks is significantly influenced by night-time care distribution (*F*(2, 2009) = 19.01, *p* < 0.001, η^2^ = 0.018) and group affiliation (*F*(1, 2009) = 730.14, *p* < 0.001, η^2^ = 0.267). Mothers primarily responsible for night-time care are also more involved in domestic tasks. Additionally, mothers in the clinical group take on significantly more domestic responsibilities than those in the control group. An interaction effect (*F*(2, 2009) = 5.22, *p* = 0.005, η^2^ = 0.005) shows that in the clinical group, unequal sharing of night-time care further amplifies mothers’ domestic burden.

#### 3.4.3. Disagreement on Educational Values

Disagreement on educational values is significantly influenced by night-time care distribution (*F*(2, 2064) = 13.99, *p* < 0.001, η^2^ = 0.013) and group affiliation (*F*(1, 2064) = 42.66, *p* < 0.001, η^2^ = 0.020). Disagreement is higher when the mother assumes sole night-time care compared to shared care (average difference = −6.02, *p* < 0.001) or father-led care (*p* = 0.007), with no significant difference between father-led and shared care (*p* = 1). Additionally, disagreement is greater in the clinical group than in the control group (average difference = −10.71, *p* < 0.001). Post hoc analyses confirm significant differences between “Shared care” and “Mother-led care” (*p* < 0.001), “Mother-led care” and “Father-led care” (*p* = 0.002), and between clinical and control groups (*p* < 0.001).

Conflict within the couple: Higher conflict levels were observed in mother-exclusive care situations (*F*(2, 2064) = 10.47, *p* < 0.001). The main group effect also shows that conflict within the couple is higher in the clinical group than in the control group (average difference = −4.00, SE = 1.64, t = −2.44, *p* = 0.015).

### 3.5. Psychopathological Variables

#### 3.5.1. Parental Burnout

No significant relationship with care distribution (*F* = 0.338, *p* = 0.261), though weak correlations were found with night-time awakenings (r = 0.09, *p* < 0.001).

#### 3.5.2. Parental Depression

Maternal depression scores were higher in cases of child insomnia (DIMS) (*F*(1, 2094) = 9.74, *p* = 0.002, R^2^ = 0.005). A slight increase in depression correlated with more awakenings (r = 0.14, *p* < 0.001).

Anxiety: Child insomnia predicted maternal anxiety (*F*(1, 1329) = 3.91, *p* < 0.05, R^2^ adjusted = 0.002). More awakenings were associated with higher anxiety (*F*(1, 1329) = 38.79, *p* < 0.001, R^2^ = 0.028).

#### 3.5.3. Parental Insomnia

A weak association existed between child and maternal insomnia (χ^2^(3) = 17.59, *p* < 0.001, V = 0.09). No significant links were found between insomnia and care distribution (χ^2^(6) = 11.06, *p* = 0.09).

## 4. Discussion

This is the first study to examine the prevalence of parental involvement in the sleep management of children aged 0 to 5 years old. All our results confirm our main hypothesis: mothers are overwhelmingly responsible for their children’s night-time care and are therefore the main players in the education and care of their children’s sleep, whether or not they have sleep problems. The presence of fathers is negligible. On average, mothers provide night-time care in 94% of cases, and fathers in 36%, meaning mothers are three times more involved than fathers. Of course, for a third of the children, fathers are as involved as mothers in this task, but this is especially true for cases in which the children sleep well. When children often wake up at night, fathers’ interventions are less frequent. As for fathers who exclusively take on the responsibility for night-time care, they exist but are rare. The differences between men and women persist in this invisible night work.

### 4.1. Parent-Related Factors of Influence

Our analyses did not identify any influencing factors that contradict this trend. This finding suggests that the inequality in night-time caregiving between fathers and mothers is primarily a societal issue rather than a biological determinant or a constraint related to the family situation.

*Professional occupation.* Our results show that mothers continue to take on the majority of night-time care, whatever their employment status. This unequal distribution persists even when mothers work full-time, illustrating the resistance of traditional parental roles to societal changes [35]. However, one limitation of our analysis is that employment status was assessed without considering work schedules (e.g., day-time vs. night-time work). The timing of professional activity may have a significant impact on night-time availability. A parent working night-shifts, for instance, may be less able to respond to night-time awakenings, independently of whether they work full- or part-time. As such, our results related to employment status should be interpreted with caution, as they do not fully capture the temporal dimension of professional obligations. Although professional occupation was significantly associated with the distribution of night-time caregiving, the variable of night-shift work was not analyzed separately due to the small number of parents concerned. Specifically, in our total sample, only 8 mothers and 14 fathers reported working night-shifts. While this may reflect a slightly higher prevalence of night work among fathers, it does not appear sufficient to explain the overall gender disparity in night-time care. Moreover, the distribution of caregiving responsibilities did not significantly differ between parents who worked night-shifts and those who did not. This suggests that working night hours does not, on its own, account for the persistent maternal overload observed in our data.

This distribution can have a direct impact on mothers’ well-being, by imposing an additional emotional and physical burden on them, amplified in the event of their child having a sleep disorder. Greater paternal involvement could be one way of reducing this unevenly distributed burden and strengthening active co-parenting [36].

*Parents’ level of education.* The influence of parents’ level of education on their night-time involvement in childcare reveals a tendency for mothers with lower levels of education to assume sole responsibility for managing night-time awakenings. This finding could be partly explained by differences in perceptions of parenting skills and by cultural beliefs related to gender roles [24]. Although mothers with higher education qualifications are more likely to share night-time care with fathers, especially in the control group, the fathers’ investment remains relatively low, even among more highly educated parents. Parents with a higher level of education tend to adopt more egalitarian beliefs and more assertive perceptions of competence [37], which promotes more balanced co-parenting. However, our results show that this distribution is never equal, indicating that additional factors could hinder fully shared care.

### 4.2. Child-Related Factors of Influence

*Age of the child.* Our results show that only 7% of fathers of children over 3 years of age take responsibility for night-time care. This result is well below the 16% reported for the same age category in a previous report [19]. This difference could be explained by the fact that our clinical and control samples both include children with sleep disorders, thus requiring frequent intervention, often assumed by the mother. Indeed, Challamel [1] points out that mothers are more inclined to manage the night-time awakenings of children in need of reassurance, a role that may be perceived as an extension of their caregiving responsibility.

This finding highlights the importance of greater paternal involvement in lightening the maternal load, particularly for children with sleep disorders.

*Rank in the sibling group order.* In our clinical sample, 96.8% of the children were only children. This strong predominance may reflect a frequent clinical demand among first-time parents, who are more likely to seek support when facing sleep-related difficulties. It also likely reflects a higher level of parental anxiety and overinvestment associated with a first child, particularly among mothers. Prior research suggests that first-time parents, and especially mothers, tend to assume a heightened caregiving role during the night, driven by both psychological and social expectations [13,21]. This may explain the very limited paternal involvement observed in our sample. While it is plausible that, in families with multiple children, caregiving duties—especially for older siblings—may be more evenly distributed between both parents, our data do not allow for a direct comparison due to the very low number of multi-child families in the clinical group. Future research should explore whether paternal night-time involvement increases with parenting experience or the presence of older siblings, which could contribute to a more balanced distribution of care.

*Child feeding method.* Analysis of the data reveals that the feeding method has a significant impact on parental involvement at night, with the majority of mothers taking charge of waking during the breastfeeding period. This result is in line with previous studies [21] showing that breastfeeding, due to the direct demand for maternal care, is associated with low paternal involvement. Furthermore, the work of Mindell and Owens [38] indicates that the closeness fostered by night-time breastfeeding creates a strengthened attachment between mother and child, often prompting mothers to respond more quickly to night-time awakenings. However, while this dynamic may be understandable in the early months, our data suggest that, even after breastfeeding ends, fathers do not become more involved in managing night-time care, perpetuating an asymmetry in parental involvement [20,21].

*Number of night-time awakenings.* Our study reveals an increase in maternal involvement in childcare when the number of night-time awakenings is high. This phenomenon may be exacerbated by societal expectations, whereby the mother’s responsibility for night-time care is often perceived as a norm. Fathers, on the other hand, are more involved when there are few or no night-time awakenings. So, fathers invest in their child’s sleep when they sleep well. Encouraging a more balanced distribution of night-time responsibilities, particularly in households with children with sleep disorders, could contribute to a better balance of parenting tasks and reduce overall parental stress [5] as well as limiting the risks of postpartum depression [16] while also improving the mother’s sleep [14].

This raises the question of causality: do fathers help mothers at night mainly when the child sleeps well, or does the father’s involvement at night reduce their child’s sleep problems?

*Sleep disorders.* The results of this study show different dynamics between the two groups. In the clinical group, a significant association was found between the distribution of night-time care and the presence of insomnia as measured by the DIMS score. More specifically, a shared distribution of responsibilities between parents was associated with the absence of insomnia, while maternal care was predominantly linked to the presence of insomnia in the child. This dynamic was not observed in the control group, where no significant association was detected. These results support studies indicating that a more balanced distribution of night-time responsibilities, notably with greater involvement of fathers, could reduce sleep disorders in children by reducing night-time awakenings and difficulties falling asleep [13,14,39].

*Sleep hygiene.* The results of our study, supported by the literature, show that parental beliefs play a crucial role in establishing night-time practices influencing the child’s autonomy in falling asleep. The high SHSC scores observed in mothers primarily responsible for night-time care may reflect limit-related cognitions that influence the frequency of night-time awakenings [4,40]. Difficulties in setting limits, particularly in response to the child’s crying, reinforce frequent nocturnal interventions, thus limiting the child’s self-soothing [9]. In comparison, fathers, who are less sensitive to their children’s night-time crying, tend to adopt an approach that encourages autonomy by setting limits, which can help reduce the child’s night-time awakenings and insomnia [15,21,41]. Their involvement therefore plays an important role not only in promoting independent sleep, but also in reducing the burden on mothers, which has positive repercussions on maternal well-being [5,14]. Our results thus reinforce the importance of encouraging a balanced distribution of night-time care, particularly in families in the clinical group, where maternal overload can intensify parental behaviors less conducive to child autonomy.

Taken together, these results suggest that mothers are particularly invested in their child’s sleep during the period when dependency is highest.

In line with this, it is important to note that our study focused exclusively on children aged 0 to 5 years, a developmental period during which sleep difficulties are most prevalent and parental intervention is frequently required. Beyond this age, children progressively gain autonomy in sleep initiation and maintenance, reducing the need for parental involvement at night. This contrasts with findings from the only large-scale French survey on this topic, which included children aged 4 to 20 years [19]. That study showed lower maternal predominance, with 31% of children cared for exclusively by their mothers, and 16% by their fathers. While the disparity remained, it appeared less pronounced than in our younger cohort. These findings suggest that gender inequalities in night-time care are particularly accentuated during early childhood, when children are most dependent, and may gradually decrease as children grow older and require less support during the night.

### 4.3. Variables Relating to Daily Life and Satisfaction Within the Couple

The more mothers provide night-time care alone, the more they report low marital satisfaction, high levels of disagreement over educational values, and higher rates of conflict within the couple. These observations align with the work of Meltzer and Mindell [10], which demonstrated that inequality in managing night-time awakenings directly impacts couple satisfaction. The absence of paternal support during night-time care can indeed induce a sense of injustice and isolation in mothers, contributing to relational tensions. The results of Millikovsky-Ayalon’s study [5] also suggest that the father’s presence during night-time care could reduce maternal stress and improve couple satisfaction. Thus, promoting a more equitable distribution of night-time care could not only benefit mothers’ mental health but also strengthen harmony within the couple.

The results of our study highlight the key role of marital conflicts and marital satisfaction in the division of night-time parental tasks. Our analyses reveal that mothers who primarily handle night-time care report lower marital satisfaction and higher rates of couple conflict. This finding aligns with the work of Meltzer and Mindell [10], who demonstrated that inequality in managing night-time awakenings can generate tensions within the couple and increase maternal psychological distress.

Our results also emphasize the influence of educational beliefs and household task distribution. Parents who disagree on educational values tend to have an unequal distribution of night-time responsibilities, with a greater burden falling on the mother. Moreover, the correlation between night-time caregiving and parental daytime involvement suggests that mothers often bear a higher overall parental load, increasing their stress and fatigue levels [5].

Finally, regression analyses indicate that mothers with high levels of depression and symptoms of anxiety are more likely to handle night-time caregiving alone. This observation suggests a vicious cycle where disproportionate night-time involvement contributes to maternal psychological distress, which in turn influences parental dynamics. Interventions targeting paternal involvement in night-time caregiving could help reduce this mental load and improve maternal well-being.

### 4.4. Psychopathological Variables

The link between child insomnia and parental depression and burnout scores, while moderate, aligns with studies indicating that child sleep disorders exacerbate chronic fatigue symptoms and mood disorders in parents [42]. Our results suggest that the accumulation of night-time awakenings contributes to this dynamic, increasing not only mothers’ physical load but also their emotional distress [5,21].

However, the lack of significant influence of night-time task allocation on parental depression and anxiety scores contrasts with some studies that have shown a protective effect of paternal involvement [14,16]. It is possible that other factors, such as parents’ educational beliefs or couple dynamics, play a more decisive role in regulating parental distress, as suggested by the results relating to educational values and satisfaction within the couple in our study.

### 4.5. Limitations and Avenues for Future Research

The inclusion of only mothers in this study is justified by the fact that only 2% of fathers completed the questionnaires. This observation is, in itself, sufficient to note the lack of involvement of fathers in their child’s sleep. The fact that only mothers took part in this study may induce a perception bias and an overestimation of their own involvement. However, the inter-rater agreement we estimated in an independent sample is very high.

This study could be criticized for the lack of single-parent families in our sample. In France, 10% of children under the age of 3 live in single-parent families [18], and since 92% of these are looked after exclusively by their mothers, the question of the distribution of night-time tasks does not arise. This is further evidence of gender inequality in child-rearing and sleep disorders.

Another limitation of our study lies in the low rate of exclusive breastfeeding reported in the control group, with only 3% of mothers indicating that their child was breastfed at night. This proportion is unexpectedly low and may reflect a recruitment bias or underreporting in the general population sample. Given the established link between breastfeeding and increased maternal involvement in night-time care, it is reasonable to assume that a higher rate of breastfeeding would have further accentuated the disparities observed between mothers and fathers. Future studies should aim to better represent breastfeeding practices, particularly in early infancy, to more accurately assess their influence on caregiving dynamics.

A further limitation herein is the absence of data regarding fathers’ mental health and behavioral well-being. While maternal variables were prioritized due to their higher response rate and their predominant role in night-time caregiving, the lack of information on paternal psychological factors limits our ability to explore their potential influence on caregiving distribution and family dynamics. Future studies should aim to include both parents to provide a more comprehensive understanding of parental mental health in relation to child sleep management.

Objective measurements of children’s sleep using actimetrics combined with a longitudinal design would provide a more reliable assessment of their sleep and the impact of fathers’ involvement thereupon. As our study is cross-sectional, it does not allow for us to determine causal links. Thus, it was not possible to determine whether fathers were more involved when the child slept well, or whether their intervention was beneficial. Only two longitudinal studies have been carried out that objectively show the positive night-time involvement of fathers, and these only studied children under six months of age, without sleep disorders, using small samples (n < 60) [14,21]. They did not report the mother–father distribution of care.

As our sample is drawn entirely from the French population, the findings must be interpreted within the context of French societal norms and gender roles. Cultural values shape parenting practices, and the persistence of maternal predominance in night-time care may be more pronounced in France than in other countries with more egalitarian parental models. Therefore, caution is required when generalizing these results to other cultural contexts. Future cross-cultural studies are needed to explore whether similar patterns of gender disparity in night-time caregiving exist elsewhere.

## 5. Conclusions

This study reveals a persistent inequality in the night-time care of young children, where mothers remain primarily responsible, regardless of their professional situation or the child’s specific needs. Although observed by clinicians specialized in child sleep, this finding had never before been studied empirically. With women increasingly involved in multiple roles in society [17], inequalities in the distribution of domestic tasks and childcare remain marked, and this study shows that responsibility for educating young children in sleep still lies predominantly with mothers. Our results also underline the fact that this disproportionate burden can affect mothers’ well-being as well as satisfaction within the couple, reinforcing the need for more balanced co-parenting. Accordingly, clinical interventions and public policies encouraging fathers to become more involved in their children’s sleep education could not only promote children’s autonomy in falling asleep, but also contribute to a more harmonious family balance, lightening the psychological load on mothers. Encouraging fathers to establish appropriate bedtime rituals, and to wait before intervening when the child cries, would help to relieve mothers and help children learn to sleep properly.

## Figures and Tables

**Table 1:** Descriptive statistics for parent-related influence variables.

	Clinical	Control
**Average age (in years)**		
Fathers	35.7	36.5
Mothers	34	34.1
**Marital status**		
In a couple or married	95%	92.48%
Living alone	5%	7.52%
**Mothers’ professional occupation**		
Full-time	41.5%	53.6%
Part-time	28.6%	21%
Unemployed	16.6%	16.3%
Maternity or parental leave	13.2%	7.5%
**Fathers’ professional occupation**		
Full-time	81.5%	85.4%
Part-time	5.8%	5%
Unemployed	2.9%	6.4%
Paternity or parental leave	0.8%	0%
**Mother’s level of education**		
Degree	87.2%	69.7%
High school diploma or vocational equivalent	8.1%	20.1%
Junior high school diploma, vocational diploma	4.3%	9.1%
No qualifications	0.3%	1.1%
**Father’s level of education**		
Degree	33.8%	47.8%
High school diploma or vocational equivalent	3.7%	21.8%
Junior high school diploma, vocational diploma	4.3%	23.4%
No qualifications	2.4%	6.9%

**Table 2:** Descriptive statistics for child-related influence variables.

	Clinical	Control
**Age category**		
0–6 months	4.3%	0.6%
6–18 months	46.9%	25.1%
18–30 months	23.6%	25.1%
30–36 months	8.7%	12.8%
3 years+	16.4%	36.2%
**Gender**		
Female	382	678
Male	500	731
**Rank in sibling group order**		
Only child	96.8%	36.5%
Oldest	0.8%	17.2%
Younger	1.1%	22.9%
Youngest	1.2%	23.4%
**Feeding method**		
Breastfed	11.8%	3%
Bottle-fed	1.9%	6%
Diversified	86.3%	91%
**Number of night-time awakenings (average)**	2.88	1.27
**Sleep disorder (pathological SDSC)**	39.72	49.84
**Presence of insomnia (pathological DIMS)**	93.1%	45.6%
**Comorbidity of organic disorders**	37.3%	41.8%

## Data Availability

No data sharing.

## Supplementary Materials

The following supporting information can be downloaded at: https://www.mdpi.com/article/10.3390/children12040491/s1, Supplementary Data S1 (a): Contingency table of the chi-square test of the distribution of night-time care between parents according to the mother‘s professional occupation. Supplementary Data S1 (b): Contingency table of the chi-square test of the distribution of night-time care between parents according to the father‘s professional occupation. Supplementary Data S2 (a): Contingency table of the chi-square test of the distribution of night-time care between parents according to the mother‘s level of education. Supplementary Data S2 (b): Contingency table of the chi-square test of the distribution of night-time care between parents according to the father‘s level of education. Supplementary Data S3: Contingency table of the chi-square test of the distribution of night-time care between parents according to rank in the sibling group order. Supplementary Data S4: Contingency table of the chi-square test of the distribution of night-time care between parents according to feeding method. Supplementary Data S5: Contingency table of the chi-square test of the distribution of night-time care between parents according to number of awakenings.

## Abbreviations

The following abbreviations are used in this manuscript:

BDI	Beck Depression Inventory
BISC	Brief Infant Sleep Questionnaire
DIMS	Difficulty in Initiating and Maintaining Sleep
ISI	Insomnia Severity Index
PBI	Parental Burnout Inventory
SDSC	Sleep Disturbance Scale for Children
SHSC	Sleep Hygiene Scale for Children
STAI	State–Trait Anxiety Inventory
