# Supplementary material for: Gender Inequality in Managing Childhood Sleep: Which Parent Gets up at Night?
Source: Children (Basel). 2025 Apr 10;12(4):491. doi: 10.3390/children12040491 (PMC12025490; doi:10.3390/children12040491)
Supplement: Supplementary file 1 [file children-12-00491-s001.zip › children-3542875-supplementary.pdf]

Gender inequality in managing childhood sleep: Which parent gets up at night?

Agnès Breton, Florian Lecuelle, Louise Chaussoy, Madeleine Heitz, Wendy Leslie, Royce Anders, Marie-Paule Gustin, Royce Anders, Franco Patricia, Benjamin Putois

Supplementary data

Supplementary data 1 (a): Contingency table of the chi-square test of the distribution of night-time care between parents according to the mother's professional occupation.

Contingency Tables

			Mother's current occupation							
Group	Who gets up		Full–time	Sick leave	Maternity/parental leave	Night work	Part–time	Student	Unemployed	Total
Clinical	Equally shared by mother and father	Count	142.000	0.000	25.000	1.000	82.000	0.000	29.000	279.000
		Standardized residuals	3.854	NaN	−2.505	0.559	0.366	NaN	−3.348	
	Mostly the father	Count	35.000	0.000	15.000	0.000	19.000	0.000	10.000	79.000
		Standardized residuals	0.531	NaN	1.608	−0.444	−0.932	NaN	−0.976	
	Mostly the mother	Count	189.000	0.000	76.000	1.000	151.000	0.000	107.000	524.000
		Standardized residuals	−3.958	NaN	1.437	−0.271	0.195	NaN	3.738	
	Total	Count	366.000	0.000	116.000	2.000	252.000	0.000	146.000	882.000
Control	Equally shared by mother and father	Count	266.000	0.000	20.000	2.000	87.000	2.000	47.000	424.000
		Standardized residuals	4.519	−0.929	−2.620	0.173	−0.296	−1.296	−3.491	
	Mostly the father	Count	38.000	1.000	9.000	2.000	18.000	1.000	9.000	78.000
		Standardized residuals	−0.887	2.752	1.383	2.984	0.462	0.264	−1.177	
	Mostly the mother	Count	451.000	1.000	77.000	2.000	191.000	11.000	174.000	907.000
		Standardized residuals	−3.905	−0.425	1.849	−1.591	0.063	1.115	3.905	
	Total	Count	755.000	2.000	106.000	6.000	296.000	14.000	230.000	1409.000
Total	Equally shared by mother and father	Count	408.000	0.000	45.000	3.000	169.000	2.000	76.000	703.000
		Standardized residuals	5.802	−0.941	−3.541	0.419	0.090	−1.335	−4.816	
	Mostly the father	Count	73.000	1.000	24.000	2.000	37.000	1.000	19.000	157.000
		Standardized residuals	−0.632	2.416	2.456	2.035	−0.107	0.043	−1.511	
	Mostly the mother	Count	640.000	1.000	153.000	3.000	342.000	11.000	281.000	1431.000
		Standardized residuals	−5.196	−0.364	2.091	−1.461	−0.029	1.249	5.375	
	Total	Count	1121.000	2.000	222.000	8.000	548.000	14.000	376.000	2291.000

Supplementary data 1 (b): Contingency table of the chi-square test of the distribution of night-time care between parents according to the father's professional occupation.

Contingency Tables

			Father's current occupation									
Group	Who gets up		Full–time	Sick leave	Night work	Other	Part–time	Paternity/parental leave	Student	Unemployed	Unknown	Total
Clinical	Equally shared by mother and father	Count	88.000	0.000	2.000	8.000	13.000	1.000	0.000	5.000	0.000	117.000
		Standardized residuals	−2.100	NaN	0.441	−0.408	2.942	0.090	NaN	1.056	NaN	
	Mostly the father	Count	26.000	0.000	0.000	3.000	3.000	1.000	0.000	1.000	0.000	34.000
		Standardized residuals	−0.788	NaN	−0.708	0.264	0.784	1.479	NaN	0.011	NaN	
	Mostly the mother	Count	194.000	0.000	3.000	18.000	6.000	1.000	0.000	5.000	0.000	227.000
		Standardized residuals	2.443	NaN	−0.002	0.231	−3.235	−0.949	NaN	−1.003	NaN	
	Total	Count	308.000	0.000	5.000	29.000	22.000	3.000	0.000	11.000	0.000	378.000
Control	Equally shared by mother and father	Count	389.000	4.000	1.000	0.000	16.000	0.000	1.000	13.000	0.000	424.000
		Standardized residuals	4.437	1.231	−1.246	NaN	−1.354	NaN	−0.914	−3.345	−3.102	
	Mostly the father	Count	66.000	0.000	0.000	0.000	9.000	0.000	0.000	3.000	0.000	78.000
		Standardized residuals	−0.197	−0.687	−0.729	NaN	2.748	NaN	−0.642	−0.944	−1.144	
	Mostly the mother	Count	748.000	4.000	8.000	0.000	45.000	0.000	6.000	74.000	22.000	907.000
		Standardized residuals	−4.156	−0.851	1.541	NaN	−0.015	NaN	1.182	3.655	3.517	
	Total	Count	1203.000	8.000	9.000	0.000	70.000	0.000	7.000	90.000	22.000	1409.000
Total	Equally shared by mother and father	Count	477.000	4.000	3.000	8.000	29.000	1.000	1.000	18.000	0.000	541.000
		Standardized residuals	2.786	1.217	−0.723	−0.318	0.267	0.115	−0.923	−2.804	−3.110	
	Mostly the father	Count	92.000	0.000	0.000	3.000	12.000	1.000	0.000	4.000	0.000	112.000
		Standardized residuals	−0.730	−0.733	−0.971	0.913	2.753	1.936	−0.685	−0.985	−1.220	
	Mostly the mother	Count	942.000	4.000	11.000	18.000	51.000	1.000	6.000	79.000	22.000	1134.000
		Standardized residuals	−2.291	−0.792	1.179	−0.157	−1.641	−1.084	1.225	3.171	3.581	
	Total	Count	1511.000	8.000	14.000	29.000	92.000	3.000	7.000	101.000	22.000	1787.000

Supplementary data 2 (a): Contingency table of the chi-square test of the distribution of night-time care between parents according to the mother's level of education.

Contingency Tables

Group	Who gets up		Undergraduate degree	Bachelor's degree	Vocational degree	Doctorate (Bac +8)	High school diploma or vocational certificate	Master's degree	Middle school diploma	No diploma or primary education certificate	Total
			Mother's education Level								
Clinical	Equally shared by mother and father	Count	17.000	51.000	6.000	33.000	15.000	157.000	0.000	0.000	279.000
		Standardized residuals	−1.301	−0.902	−2.147	1.165	−2.056	2.855	NaN	−1.180	
	Mostly the father	Count	2.000	12.000	4.000	10.000	6.000	44.000	0.000	1.000	79.000
		Standardized residuals	−1.836	−1.135	0.346	0.794	−0.193	1.209	NaN	1.481	
	Mostly the mother	Count	50.000	114.000	28.000	46.000	51.000	233.000	0.000	2.000	524.000
		Standardized residuals	2.300	1.514	1.832	−1.565	2.060	−3.407	NaN	0.256	
	Total	Count	69.000	177.000	38.000	89.000	72.000	434.000	0.000	3.000	882.000
Control	Equally shared by mother and father	Count	88.000	105.000	22.000	122.000	81.000	0.000	5.000	1.000	424.000
		Standardized residuals	−0.782	1.775	−2.348	1.695	−0.603	NaN	−0.500	−2.091	
	Mostly the father	Count	15.000	18.000	2.000	27.000	11.000	0.000	3.000	2.000	78.000
		Standardized residuals	−0.623	0.284	−1.759	1.839	−1.357	NaN	1.864	1.225	
	Mostly the mother	Count	208.000	184.000	85.000	214.000	191.000	0.000	12.000	13.000	907.000
		Standardized residuals	1.047	−1.836	3.089	−2.502	1.226	NaN	−0.411	1.418	
	Total	Count	311.000	307.000	109.000	363.000	283.000	0.000	20.000	16.000	1409.000
Total	Equally shared by mother and father	Count	105.000	156.000	28.000	155.000	96.000	157.000	5.000	1.000	703.000
		Standardized residuals	−1.413	0.830	−3.163	1.856	−1.619	2.754	−0.554	−2.413	
	Mostly the father	Count	17.000	30.000	6.000	37.000	17.000	44.000	3.000	3.000	157.000
		Standardized residuals	−2.010	−0.642	−1.375	1.252	−1.675	3.009	1.448	1.548	
	Mostly the mother	Count	258.000	298.000	113.000	260.000	242.000	233.000	12.000	15.000	1431.000
		Standardized residuals	2.395	−0.456	3.729	−2.421	2.416	−4.193	−0.228	1.490	
	Total	Count	380.000	484.000	147.000	452.000	355.000	434.000	20.000	19.000	2291.000

Supplementary data 2 (b): Contingency table of the chi-square test of the distribution of night-time care between parents according to the father's level of education.

Contingency Tables ▼

Group	Who gets up		Undergraduate degree	Bachelor's degree	Vocationnal degree	Doctorate (Bac +8)	High school diploma or vocational certificate	Master's degree	Middle school diploma	No diploma or primary education certificate	Total
			Father's education level								
Clinical	Equally shared by mother and father	Count	17.000	23.000	11.000	7.000	7.000	52.000	0.000	0.000	117.000
		Standardized residuals	0.395	1.246	−0.282	−0.055	−1.267	0.348	NaN	−2.033	
	Mostly the father	Count	1.000	6.000	3.000	4.000	2.000	18.000	0.000	0.000	34.000
		Standardized residuals	−1.888	0.251	−0.250	1.452	−0.617	1.212	NaN	−0.955	
	Mostly the mother	Count	33.000	32.000	24.000	12.000	24.000	93.000	0.000	9.000	227.000
		Standardized residuals	0.729	−1.322	0.412	−0.796	1.556	−1.036	NaN	2.476	
	Total	Count	51.000	61.000	38.000	23.000	33.000	163.000	0.000	9.000	378.000
Control	Equally shared by mother and father	Count	89.000	58.000	52.000	106.000	91.000	0.000	14.000	14.000	424.000
		Standardized residuals	2.391	1.864	−5.067	3.603	−0.237	NaN	0.687	−3.485	
	Mostly the father	Count	15.000	13.000	7.000	23.000	14.000	0.000	3.000	3.000	78.000
		Standardized residuals	0.460	1.546	−2.609	2.364	−0.860	NaN	0.551	−1.090	
	Mostly the mother	Count	140.000	88.000	231.000	142.000	203.000	0.000	23.000	80.000	907.000
		Standardized residuals	−2.509	−2.523	6.098	−4.580	0.637	NaN	−0.921	3.858	
	Total	Count	244.000	159.000	290.000	271.000	308.000	0.000	40.000	97.000	1409.000
Total	Equally shared by mother and father	Count	106.000	81.000	63.000	113.000	98.000	52.000	14.000	14.000	541.000
		Standardized residuals	2.315	2.256	−4.828	3.332	−0.686	0.474	0.658	−3.943	
	Mostly the father	Count	16.000	19.000	10.000	27.000	16.000	18.000	3.000	3.000	112.000
		Standardized residuals	−0.654	1.548	−2.662	2.257	−1.334	2.639	0.325	−1.505	
	Mostly the mother	Count	173.000	120.000	255.000	154.000	227.000	93.000	23.000	89.000	1134.000
		Standardized residuals	−1.879	−2.932	5.946	−4.315	1.326	−1.781	−0.791	4.520	
	Total	Count	295.000	220.000	328.000	294.000	341.000	163.000	40.000	106.000	1787.000

Supplementary data 3: Contingency table of the chi-square test of the distribution of night-time care between parents according to rank in the sibling group order.

Contingency Tables ▼

Group	Who gets up		Siblings 0	1	2	3	Total
Clinical	Equally shared by mother and father	Count	268.000	2.000	5.000	4.000	279.000
		Standardized residuals	−0.885	−0.175	1.256	0.340	
	Mostly the father	Count	74.000	3.000	1.000	1.000	79.000
		Standardized residuals	−1.676	3.153	0.116	0.016	
	Mostly the mother	Count	512.000	2.000	4.000	6.000	524.000
		Standardized residuals	1.813	−1.668	−1.257	−0.331	
	Total	Count	854.000	7.000	10.000	11.000	882.000
Control	Equally shared by mother and father	Count	158.000	87.000	105.000	74.000	424.000
		Standardized residuals	0.401	2.133	1.121	−3.471	
	Mostly the father	Count	21.000	22.000	19.000	16.000	78.000
		Standardized residuals	−1.804	2.636	0.326	−0.624	
	Mostly the mother	Count	335.000	134.000	198.000	240.000	907.000
		Standardized residuals	0.477	−3.302	−1.229	3.622	
	Total	Count	514.000	243.000	322.000	330.000	1409.000
Total	Equally shared by mother and father	Count	426.000	89.000	110.000	78.000	703.000
		Standardized residuals	0.575	1.785	1.046	−3.390	
	Mostly the father	Count	95.000	25.000	20.000	17.000	157.000
		Standardized residuals	0.211	2.087	−0.646	−1.480	
	Mostly the mother	Count	847.000	136.000	202.000	246.000	1431.000
		Standardized residuals	−0.658	−2.789	−0.659	4.001	
	Total	Count	1368.000	250.000	332.000	341.000	2291.000

Supplementary data 4: Contingency table of the chi-square test of the distribution of night-time care between parents according to feeding method.

Contingency Tables ▼

Group	Who get's up simplified		Current main feeding method: Breastfeeding 0 / Bottle-feeding 1 / Diversified 2 — Bottle-feeding	Breastfeeding	Diversified	Total
Clinical	Equally shared by mother and father	Count	9.000	19.000	251.000	279.000
		Standardized residuals	1.908	−3.120	2.163	
	Mostly the father	Count	0.000	5.000	74.000	79.000
		Standardized residuals	−1.306	−1.578	2.001	
	Mostly the mother	Count	8.000	80.000	436.000	524.000
		Standardized residuals	−1.047	3.872	−3.211	
	Total	Count	17.000	104.000	761.000	882.000
Control	Equally shared by mother and father	Count	28.000	6.000	390.000	424.000
		Standardized residuals	0.591	−2.267	0.855	
	Mostly the father	Count	7.000	3.000	68.000	78.000
		Standardized residuals	1.123	0.462	−1.208	
	Mostly the mother	Count	50.000	33.000	824.000	907.000
		Standardized residuals	−1.102	1.951	−0.242	
	Total	Count	85.000	42.000	1282.000	1409.000
Total	Equally shared by mother and father	Count	37.000	25.000	641.000	703.000
		Standardized residuals	1.252	−3.672	2.056	
	Mostly the father	Count	7.000	8.000	142.000	157.000
		Standardized residuals	0.004	−0.679	0.531	
	Mostly the mother	Count	58.000	113.000	1260.000	1431.000
		Standardized residuals	−1.195	3.852	−2.235	
	Total	Count	102.000	146.000	2043.000	2291.000

Supplementary data 5: Contingency table of the chi-square test of the distribution of night-time care between parents according to number of awakenings.

Contingency Tables

			Number of awakenings										
Group	Who gets up		0	1	2	3	4	5	6	7	8	10	Total
Clinical	Equally shared by mother and father	Count	30.000	67.000	67.000	46.000	27.000	21.000	7.000	5.000	6.000	3.000	279.000
		Standardized residuals	0.523	3.557	0.151	−0.279	−1.389	−0.629	−2.975	−1.034	1.940	−1.252	
	Mostly the father	Count	11.000	16.000	26.000	15.000	5.000	2.000	1.000	2.000	0.000	1.000	79.000
		Standardized residuals	1.227	0.715	2.019	0.491	−1.604	−1.968	−1.859	−0.044	−0.998	−0.448	
	Mostly the mother	Count	47.000	70.000	116.000	89.000	73.000	51.000	45.000	16.000	4.000	13.000	524.000
		Standardized residuals	−1.208	−3.784	−1.317	−0.021	2.248	1.740	3.899	1.005	−1.257	1.446	
	Total	Count	88.000	153.000	209.000	150.000	105.000	74.000	53.000	23.000	10.000	17.000	882.000
Control	Equally shared by mother and father	Count	169.000	141.000	61.000	37.000	8.000	5.000	0.000	0.000	1.000	2.000	424.000
		Standardized residuals	3.945	−0.859	−2.457	−0.247	−1.138	−0.500	−1.975	−0.929	1.525	−0.315	
	Mostly the father	Count	24.000	32.000	16.000	4.000	1.000	1.000	0.000	0.000	0.000	0.000	78.000
		Standardized residuals	−0.310	1.164	0.535	−1.233	−0.764	−0.106	−0.729	−0.343	−0.242	−0.687	
	Mostly the mother	Count	263.000	319.000	180.000	86.000	28.000	14.000	9.000	2.000	0.000	6.000	907.000
		Standardized residuals	−3.631	0.267	2.098	0.825	1.455	0.529	2.239	1.053	−1.345	0.629	
	Total	Count	456.000	492.000	257.000	127.000	37.000	20.000	9.000	2.000	1.000	8.000	1409.000
Total	Equally shared by mother and father	Count	199.000	208.000	128.000	83.000	35.000	26.000	7.000	5.000	7.000	5.000	703.000
		Standardized residuals	3.414	1.015	−1.687	−0.278	−1.611	−0.650	−3.357	−1.165	2.375	−1.165	
	Mostly the father	Count	35.000	48.000	42.000	19.000	6.000	3.000	1.000	2.000	0.000	1.000	157.000
		Standardized residuals	−0.443	0.698	2.068	0.004	−1.280	−1.435	−1.656	0.228	−0.902	−0.568	
	Mostly the mother	Count	310.000	389.000	296.000	175.000	101.000	65.000	54.000	18.000	4.000	19.000	1431.000
		Standardized residuals	−3.021	−1.331	0.528	0.262	2.202	1.367	4.061	0.990	−1.792	1.406	
	Total	Count	544.000	645.000	466.000	277.000	142.000	94.000	62.000	25.000	11.000	25.000	2291.000
